# Diabetes and Sarcopenic Obesity: Pathogenesis, Diagnosis, and Treatments

**DOI:** 10.3389/fendo.2020.00568

**Published:** 2020-08-25

**Authors:** Mina Wang, Yan Tan, Yifan Shi, Xu Wang, Zehuan Liao, Peng Wei

**Affiliations:** ^1^School of Traditional Chinese Medicine, School of Life Sciences, Beijing University of Chinese Medicine, Beijing, China; ^2^Beijing Key Laboratory of Acupuncture Neuromodulation, Department of Acupuncture and Moxibustion, Beijing Hospital of Traditional Chinese Medicine, Capital Medical University, Beijing, China; ^3^School of Biological Sciences, Nanyang Technological University, Singapore, Singapore; ^4^Department of Microbiology, Tumor and Cell Biology (MTC), Karolinska Institutet, Biomedicum, Stockholm, Sweden

**Keywords:** diabetes, sarcopenic obesity, insulin resistance, aging, inflammation

## Abstract

Sarcopenic obesity and diabetes are two increasing health problems worldwide, which both share many common risk factors, such as aging, and general obesity. The pathogenesis of sarcopenic obesity includes aging, physical inactivity, malnutrition, low-grade inflammation, insulin resistance, and hormonal changes. Nevertheless, there are two major reasons to cause diabetes: impaired insulin secretion and impaired insulin action. Furthermore, the individual diagnosis of obesity and sarcopenia should be combined to adequately define sarcopenic obesity. Also, the diagnosis of diabetes includes fasting plasma glucose test (FPG), 2-h oral glucose tolerance test (OGTT), glycated hemoglobin (A1C), and random plasma glucose coupled with symptoms. Healthy diet and physical activity are beneficial to both sarcopenic obesity and diabetes, but there are only recommended drugs for diabetes. This review consolidates and discusses the latest research in pathogenesis, diagnosis, and treatments of diabetes and sarcopenic obesity.

## Introduction

There are two greatest epidemiological trends of world—aging and obesity with the extension of average lifetime span and the changing lifestyle. The two factors dramatically affect body composition, morbidity and mortality (1). Aging is associated with the decrease in muscle mass and strength and the increase in body fat mass, which leads to frailty, falls, disability, social isolation, and hospitalization. The word sarcopenia from Greek refers to age-related loss of muscle mass. Although the impact of sarcopenia has been well-demonstrated, the effect of obesity on it emerges as a new public health problem (2). Therefore, a new term sarcopenic obesity (SO) arises to represent the coexistence of sarcopenia and obesity (3). Compared to simple sarcopenia or obesity alone, the medical sequelae related to SO are much greater, causing significantly higher healthcare costs (4). Data from Health and Nutrition Examination Survey (NHANES) noted SO rates of 12.6% in men and 33.5% in women (5). With the rapid growth of the elderly population globally, it was estimated that SO would affect 100–200 million people from 2016 to 2051 (6). Although SO is more common in older people due to the natural changes in body composition, it is estimated that younger people with class II or III obesity are vulnerable as well. A study assessed the prevalence of SO in 120 young adults using different diagnostic criteria of SO, revealing that 23.3% female and 58.8% male suffered SO according to the definition of appendicular skeletal muscle mass (ASM) by weight × 100 % (7). Due to the chronic progress of SO, the symptoms hardly draw people's attention, resulting in poor diagnosis, which causes negative consequences of quality of life and all-cause mortality (8).

**Figure 1:**
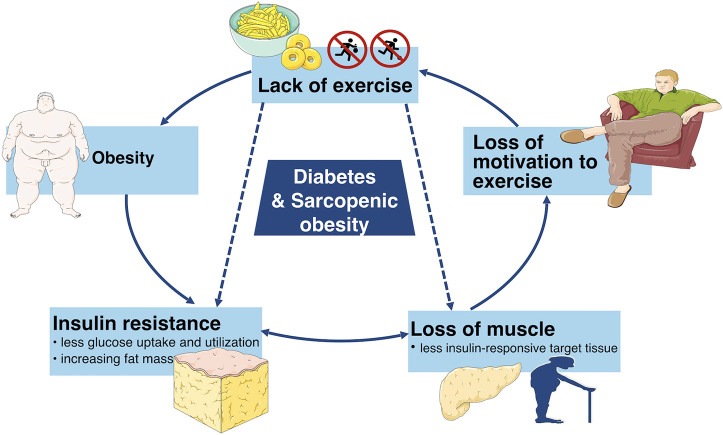
This simplified schematic diagram depicts the vicious cycle of unhealthy lifestyle which can eventually lead to diabetes and sarcopenic obesity as well as other adverse metabolic conditions.

Moreover, aging and obesity are two risk factors of diabetes as well. A variety of studies have the consensus that aging as well as obesity have positive associations with diabetes. A study in America has suggested that the percentage of diabetics rises with age (9). Furthermore, a study has estimated that the number of global diabetic patients would increase from 422 million in 2014 to at least 592 million in 2,035 and it has been shown that more than 50% of the diabetics are obese (10). Hence, there is a good reason to suspect that diabetes and SO have a strong relationship (11). On the one hand, insulin loses the function to enhance cellular glucose uptake and utilization in diabetics, which is defined clinically as insulin resistance which promotes obesity. Furthermore, the increasing fat mass facilitates various cytokines which accelerate the catabolism of muscles. On the other hand, the loss of muscle mass leads to less insulin-responsive target tissue, resulting in a severe condition of insulin resistance (12, 13). Therefore, the vicious cycle continues until more negative health consequences occur (Figure 1). Hence, it is necessary to deduce the pathogenesis, diagnosis and treatments of both diabetes and SO in order to stop the vicious cycle and increase the quality of life. This review consolidates and discusses the latest research in pathogenesis, diagnosis and treatments of diabetes, and sarcopenic obesity.

## Pathogenesis and Complications of Sarcopenic Obesity

### Pathogenesis of Sarcopenic Obesity

There are multiple factors that cause sarcopenic obesity, such as aging, lack of physical activity, malnutrition low-grade inflammation, insulin resistance and hormonal changes, which leads to body composition changes (muscle mass and strength decline and fat mass increase). Moreover, each factor has independent impacts on the quality and quantity of muscle and fat, whereas, the cross-talks of them have stronger influences.

#### Aging

With aging, the reduction of basal metabolic rates may lead to weight gain and muscle mass decrease, which are associated with SO. Indeed, young people generally have more bone mass and muscle strength than old people. Meanwhile, studies have validated that the amount of bone mass and muscle mass peak around 30 years old, and after that, a gradual loss of muscle mass is accompanied with a parallel gain of fat mass (14). Therefore, weight generally increases in elderly people. However, it is also common that SO occurs in the old without increase of body weight, which could be due to the ectopic redistribution of fat. Studies have found that fat tends to move to viscera, muscle and abdomen with age, and the ectopic fat cause disorders of inflammatory factors, insulin and hormones, thus resulting in SO (15).

#### Lack of Physical Activity

Age-related SO is usually due to the lack of physical activity. In fact, old people tend to decrease physical activity, which contributes to the severe loss of muscle strength. Then atrophic muscles make even more difficult for elderly people to exercise, further aggravating the sedentary lifestyle. Numerous studies have proven that physical activity is necessary to lose weight and improve muscle strength, which is beneficial to ameliorate the state of SO (16, 17).

#### Imbalanced Nutrition

The imbalance of energy intake and expenditure is also linked to SO, especially for the old. On one hand, old people obtain inadequate protein in their diet. It is difficult to sustain muscle mass and strength with insufficient amount of protein intake (18). On the other hand, owing to decrease of outdoor physical activities, vitamin D produces less with scanty expose of ultraviolet radiation (19). Hence, inadequate vitamin D accelerates SO (20).

#### Low-Grade Inflammation

Both aging and physical inactivity have contributions on weight gain, by which an increase in adipose cell size may be determined. Specifically, adipocytes promote macrophage recruitment (21), then adipocytes and immune cells secrete more adipokines such as leptin, chemerin, resistin, and more cytokines such as tumor necrosis factor-α (TNF-α), interleukins (ILs), interferon-γ (INF-γ) (22–25), creating a circumstance of low-grade inflammation. Previous studies have indicated that an inflammatory state plays a significant role in the progression of SO as well as the morbidity and mortality driven by SO. A study conducted in Italy has verified that elevated levels of IL-6 and C-reactive protein (CRP) are related to SO, which in turn have suppressive effects on muscle strength (26). Moreover, a similar result has been shown in another cross-sectional study. The study has found the highest level of monocyte chemoattractant protein-1 (MCP-1) and the lowest level of appendicular lean mass in SO group (27), which supports the theory that low-grade inflammation is linked to SO.

Besides, the increased level of leptin contributes to leptin resistance, resulting in accumulation of free fatty acids. As less amount of fatty acid are oxidized in muscles, more fat mass deposits in visceral such as liver, heart, and spleen. Thereby, dysfunctional insulin production occurs, gradually leading to insulin resistance (28). Furthermore, the reduction of fatty acid consumption is coupled with the increase of reactive oxygen species generation. Oxidative stress is strongly associated with the expression of various inflammatory transcription factors, such as nuclear factor-kB (NF- kB) that modulates proteolytic pathways and promotes inflammation (29–31). Moreover, either low-grade inflammation or ectopic fat distribution generates myokine imbalance and mitochondrial dysfunction (32, 33). Particularly, myokines can exacerbate insulin resistance. Also, mitochondrial dysfunction causes more lipid peroxidation, which reinforces the collections of lipid intermediates and reactive oxygen metabolites, accelerating inflammation, insulin resistance, and oxidative stress (33). Hence, another vicious circle leading to SO forms.

#### Insulin Resistance

The production and efficiency of insulin decline in elderly and obese people. Meanwhile, obesity is related to a low-grade inflammation, the increased production and secretion of multifarious inflammatory factors including TNF-α, IL-6 modulate insulin sensitivity by altering some key steps in the insulin signaling pathway, which is responsible for the subsequent insulin resistance (34–36). Studies have elucidated that insulin resistance is essential for protein anabolism, thus directly concerns muscle fiber atrophy (37). Obese individuals with insulin resistance have a higher rate of muscle catabolism, which has been evidenced in a study that leg muscle strength and quality decrease distinctly in older diabetics (38). Therefore, insulin resistance is involved in poor muscle mass and muscle strength, progressively resulting in SO. Furthermore, insulin resistance is correlated to mitochondrial dysfunction as well. A down-regulation of genes involving mitochondrial enzymes declines mitochondrial content, which has been found in insulin resistant states (39, 40), augmenting accumulation of fat in muscle and liver. Hence, the loss of muscle strength and the gain of fat which characterize SO is attributed by insulin resistance (41).

#### Hormonal Changes

As an endocrine organ, the muscle can produce a variety of myokines such as myostatin and irisin. It is believed that myostatin inhibits muscle cell growth and differentiation, and irisin stimulates the increase of muscle mass (42, 43). However, several studies have documented that the content of myostatin is upregulated while irisin is downregulated in sarcopenia (44). At the same time, the increase in myostatin and decrease in irisin are tightly associated with poor browning reaction of white fat, reducing energy expenditure, triggering fat gain (45). Eventually, the crosstalk between muscle and fat leads to muscle damage, eliciting SO.

Other hormones, including insulin-like growth factors-1 (IGF-1), growth hormone, testosterone, and estrogen, also regulate the anabolic and catabolic progressions in muscle (46, 47). The reduction of IGF-1 is accompanied by the downregulation of irisin (45), and high level of free fatty acids in obese people inhibits both IGF-1 and growth hormone (48), which lowers the mass and strength of muscle, leading to muscle impairment and thus to SO (49). Moreover, testosterone and estrogen are essential for muscle health (47), but the production of these hormones decline naturally with aging. Hence, the muscle mass and strength weaken with the reduced testosterone and estrogen concentrations (50). Therefore, aberrant hormonal changes with age exacerbate SO.

### Complications of Sarcopenic Obesity

It is acknowledged that either low muscle mass and strength or obesity is an independent risk factor for reduced physical capacity and quality of life. Therefore, it is reasonable to speculate when muscle damage and obesity coexist, they act synergistically on the risk of mortality, metabolic disorders, and quality of life (47, 51). Firstly, SO is overtly associated with an increased risk of all-cause mortality. A meta-analysis of 12 prospective cohort studies has indicated that compare to non-SO participants, subjects with SO have a 24% increased risk of all-cause mortality, especially in men. Noteworthily, according to different definition of SO, the risk of all-cause mortality changes. Specifically, all-cause mortality is higher basing on the criteria of mid-arm muscle circumference or muscle strength (HR 1.46, 95% CI 1.23–1.73 and 1.23, 1.09–1.38, respectively) rather than on the definition of skeletal muscle mass (pooled HR 1.24, 95% CI 1.12–1.37, *P* < 0.001) (52). Moreover, in a Japanese study, all-cause mortality increases in men with SO defined by waist circumference (HR, 1.19; 95% CI, 1.02–1.38), but not body mass index (BMI) or percent body fat (BF%) (53). Secondly, metabolic disorders including cardiometabolic syndrome (CMS), diabetes, cardiovascular disease (CVD), and cancer are common comorbidities of SO (54, 55). Several Korean studies have indicated that individuals with SO are associated with increased waist circumference, elevated fasting blood glucose, insulin resistance, higher blood pressure, and abnormal blood lipids as compared to sarcopenia or obesity alone (13, 23, 56). The cluster of abdominal obesity, hyperglycemia, hyperinsulinemia, dyslipidemia, and hypertension that composes CMS progressively leads to diabetes and CVD. A meta-analysis including 606 articles has demonstrated that SO increases the risk of T2D by almost 38% compared to individuals with excess weight or obesity alone (11). Furthermore, the characteristic may attribute to physical disability and metabolic syndromes, which has been clarified in another Korean study (57). In addition, studies have also estimated 10-years risk of CVD in SO group, non-sarcopenic group, and non-obese group, and a significant increase has been found in the SO group (58). Moreover, adverse clinical cancer outcomes reported to be relevant to SO, especially with respect to dose-limiting toxicity, surgical complications, physical disability, and shorter survival (59–61). Finally, even if there is no discrepancies between SO group and other groups of physical capacity found in a study (62), most studies support that SO causes physical disability. As the loss of muscle mass and strength makes a higher risk of osteoarthritis (63, 64), this exacerbates physical inactivity, worsening both physical and mental health. A cross-sectional study has evaluated the association between SO and cognitive function in geriatric population, and found that SO is an indicator of probable cognitive impairment (65). Therefore, these factors, which significantly affect the quality of life for elderly people, have been illustrated in another study that the highest fall risk and the lowest muscle function test results in the SO group, confirming an inverse association with health-related quality of life scores (66).

## Pathogenesis and Complications of Diabetes

### Pathogenesis of Diabetes

Diabetes mellitus is a chronic metabolic disease involving persistently elevated levels of blood glucose. Diabetes can be classified into several types: type 1 diabetes (T1D), type 2 diabetes (T2D), gestational diabetes, maturity-onset diabetes of the youth, neonatal diabetes, and secondary diabetes resulting from endocrinopathies, steroid use. The major subtypes of diabetes are T1D and T2D (67, 68). In 1889, scientists first discovered the role of the pancreas in the pathogenesis of diabetes. There are two main subtypes of endocrine cells in the pancreatic islets: β cells and α cells. β cells are involved in the production insulin, while α cells are responsible for secreting glucagon. The function of β cells and α cells changes with the glucose environment in an individual's body (69, 70). Once the imbalance between the secretion of insulin and glucagon occurs, the levels of blood glucose skew improperly as well. In the case of diabetes, it may be linked to impaired insulin secretion (insulin deficiency), impaired insulin action (insulin resistance), or both (71).

#### Impaired Insulin Secretion

Impaired insulin secretion is multifactorial, the exact mechanism is still unclear, but commonly develops from glucose toxicity, lipid toxicity, immunoinflammatory response, and oxidative stress, leading to the dysfunction of islet β cells (72). Persistent elevated levels of glucose can swamp the glycolytic process and inhibit glyceraldehyde catabolism, which promotes reactive oxygen species (ROS) production and oxidative damage, inducing β cells apoptosis and inhibiting β cells secretion (73, 74).

The accumulation of free fatty acids in the islet β cells accelerates the production of NO, which causes the apoptosis of β cells. Furthermore, long chain saturated fatty acids can inhibit the expression of adenine nucleotide translocator. Thus, the inner mitochondrial membrane of islet β cells fails to protect, which increases the permeability of mitochondrial membrane, leading to β cells apoptosis (75, 76).

In fact, the chronic activation of the innate immune system, leading to intra-islet inflammation also seems to be the key part of β cells apoptosis (77). Both obesity and hyperglycemia promote the release of inflammatory mediators, like TNF-α, or IL-6, released mediators stimulate macrophages and other innate immune cells, as well as some apoptosis-related signaling pathways, such as Fas/FasL signaling pathway (78, 79). This leads to the destruction and dysfunction of certain cells, like islet β cells that produce insulin. Besides, chronic inflammatory environment is also beneficial for the formation of free radicals such as reactive oxygen species (ROS), exacerbating β cells damage and yielding a positive feedback circle with the secretion of more detrimental cytokines to trigger further damage to β cells (80).

**Figure 2:**
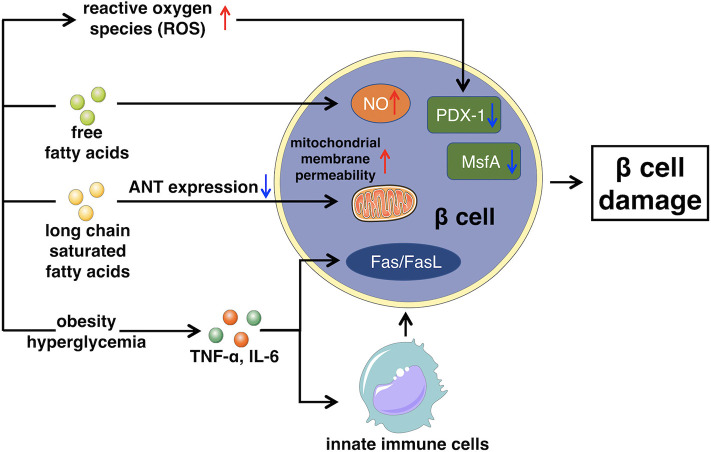
This figure illustrates the main mechanism of impaired insulin secretion that glucose toxicity, lipid toxicity, immunoinflammatory response, and oxidative stress lead to β cell damage. ANT, Adenine nucleotide translocator.

As mentioned above, prolonged as well as elevated levels of glucose blood, high free fatty acids, and chronic inflammatory environment, all increase the levels of ROS, activating the mechanism of oxidative stress (81). Furthermore, the islet β cells are especially sensitive to ROS because of their low inherent level of antioxidant enzymes. Therefore, ROS is capable to directly damage β cells, promoting apoptosis, or it can indirectly regulate insulin signaling pathway to inhibit the function of β cells (82, 83). Moreover, chronically excessive levels of ROS can cause the loss of transcription factors PDX-1 and MsfA, disturbing the expression of insulin gene (84). With the destruction of β cells, the peak of insulin secretion will be delayed, intensifying the fluctuation of blood glucose, resulting in further severe damage of β cells (85). The general mechanism of how glucose toxicity, lipid toxicity, immunoinflammatory response, as well as oxidative stress cause β cell damage, leading to impaired insulin secretion is illustrated in Figure 2.

#### Impaired Insulin Action

Impaired insulin action refers to the reduction of glucose uptake and utilization. In order to sustain a stable levels of blood glucose, islet β cells secret excessive insulin to compensate, leading to hyperinsulinemia (86). Increased insulin content results in less affinity of insulin receptor (IR) thus, cells (major muscle) gradually become insensitive to insulin. IR is a member of the ligand-activated receptor and tyrosine kinase family of transmembrane signaling proteins composed of two α- subunits and two β-subunits linked by disulfide bonds (87). The main function of the two α- subunits is to bind to insulin as they are located at the extracellular surface. While the two β-subunits are distributed at extracellular, transmembrane, and intracellular sites, which regulate insulin- stimulated tyrosine kinase activity. After the α- subunits bind to insulin, insulin receptor tyrosine kinase is activated by phosphorylating of the β-subunits on multiple tyrosine residues. The main physiological role of insulin receptor tyrosine kinase appears to be metabolic regulation. Therefore, any impairments occur on main phosphorylation sites will reduce insulin receptor tyrosine kinase activity, eventually affecting the insulin function (88–90).

Furthermore, there are multiple factors, particular obesity, increase the levels of adipocytes, inflammatory cytokines (IL-1, IL-6, and TNFα) (91). The elevated amount of these components stimulates several signaling pathways such as inhibitor kappa beta kinase beta (IKKβ), Jun N-terminal kinase (JNK), and NF-κB signaling pathways to induce the development of insulin resistance (92, 93). Meanwhile, the transduction of the insulin PI3K/AKT signaling pathway is weakened, which may be the major manifestation of insulin resistance, impacting the downstream mediator—glycogen synthase kinase-3β (GSK3β). GSK3β is one of the few protein kinases of which the activity can be inhibited by phosphorylation (94).

**Figure 3:**
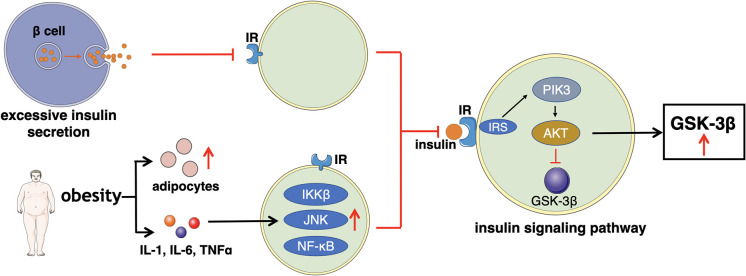
This figure illustrates the main mechanism of impaired insulin action that excessive insulin secretion, adipocytes, and inflammatory factors directly affect IR or indirectly interfere the insulin signaling pathway.

Insulin signaling pathway begins with the binding to its cell surface IR, which has a tyrosine- protein kinase activity, and regulates the insulin response (95). Then, the binding of insulin and its IR stimulates the association between the receptor and downstream mediators, such as insulin receptor substrate-1 (IRS-1) and phosphatidylinositol 3-kinase (PI3K). The insulin receptor can directly activate PI3K by binding to the p85 regulatory subunit, leading to the production of phosphatidylinositol-3,4,5-triphosphate (PIP3). Moreover, the indirect PI3K activation is associated with phosphorylation and activation of AKT, also known as protein kinase B (PKB). Afterwards, AKT inhibits GSK3β by phosphorylating at Ser9 site. When the insulin signaling pathway fails, the expression of GSK3β improves, which reduces the insulin sensitivity and increases the levels of blood glucose, thus subsequently leading to T2D (72, 96, 97). The general mechanism of how excessive insulin secretion, adipocytes, and inflammatory factors directly affect IR or indirectly interfere the insulin signaling pathway, leading to impaired insulin action is illustrated in Figure 3.

### Complications of Diabetes

Unmanaged diabetes can cause multiple complications, most common of which are microvascular complications: nephropathy, retinopathy, and neuropathy, and macrovascular complications: cardiovascular diseases.

#### Renal Disease

Diabetic kidney disease is defined as persistent albuminuria, accompanied with a persistent reduction of glomerular filtration rate and an increase of arterial blood pressure, which can progress to end-stage renal disease (ESRD) (98). The prevalence of diabetic kidney disease in patients with T1D and T2D is 30 and 40%, respectively (99). A study has found that the risk of diabetic kidney disease is much higher in Asian countries than in Western countries due to different economic outcomes (100). Furthermore, the incidence of ESRD has been estimated to be about 40%-50% in the United States (101). In fact, more than 50% of the patients in America with T1D will eventually receive renal replacement treatments (98). Besides, the prevalence of ESRD is even higher than 60% in Malaysia, Singapore, and Mexico, which may relate to genetic background, lifestyle, health awareness and economic situation (101).

#### Ocular Disease

There are many diabetes-related ocular diseases, such as cataract, glaucoma, ischemic optic neuropathy, cranial nerve palsies, and recurrent corneal erosion syndrome (102). However, diabetic retinopathy is the most well-known complication, and it is expected to increase from 415 million in 2015 to 642 million by 2040 (103). Moreover, World Health Organization (WHO) has estimated that diabetic retinopathy leads to blindness in 5% of blind people (104). One of the structural changes in diabetic retinopathy is widened retinal arteriolar caliber which might be an early sign of microvascular dysfunction. Also, another change is widened retinal venular caliber which might be independently related to the prevalence and progression in diabetic retinopathy, as well as an indicator of developing retinopathy (105, 106).

#### Diabetic Neuropathy

Diabetic neuropathy is one of the most prominent complications of diabetes. The true prevalence is unclear but it varies from 10 to 90% in diabetics, according to the different standards and approaches used to define diabetic neuropathy (107). Diabetic neuropathy involves various syndromes, such as mono- and polyneuropathies, plexopathies, radiculopathies and autonomic neuropathy, among which distal symmetric polyneuropathy is very prevalent (108). Meanwhile, distal symmetric polyneuropathy mainly contributes to disability in diabetics, stemming from gait disturbance, fall-related injury, and foot ulceration and amputation (109). It has been reported that neuropathy is associated with a 1.7-fold increase in the risk of amputation, and even higher when coupled with other problems (107). In the UK, approximately one third amputees have a history of diabetes (110), and in Australia, about half of the amputees have diabetes (111). Therefore, diabetic neuropathy extremely influences the quality of life in diabetic people.

#### Cardiovascular Diseases

Cardiovascular diseases, involving coronary heart disease, peripheral vascular disease and cerebrovascular disease, are the primary contributors of death and disability in diabetic people (112). Individuals with diabetes suffer from a higher risk of cardiovascular diseases compared to those without diabetes, which is associated with a number of common risk factors such as age, obesity, smoking, hypertension, and dyslipidemia (113). Moreover, recent studies have shown that diabetes is considered as an independent risk factor for coronary heart diseases (114). Interestingly, a study has indicated that patients with T2D in Asian countries have a lower risk of coronary diseases than those from eastern Europe or Established Market Economies (100). However, Indian patients with T2D are as twice likely to develop coronary artery diseases as white Europeans who have T2D (115).

#### Cancer

Numerous epidemiological evidences have indicated that diabetes is considered as an independent risk factor for increased rates of various types of cancer. Cancer is a class of diseases resulted from external factors such as environment, diet and radiation as well as internal factors including obesity and diabetes (31, 116, 117). It has been proven that there is 1–2% of diabetic patients who will develop pancreatic cancer in 3 years (118). Also, diabetes serves as an independent risk factor for colorectal cancer as a study has found that there is a 49% increased risk of colorectal cancer in men with diabetes (119). Moreover, diabetes contributes a 27% increased risk of breast cancer in women and predisposes to more aggressive cancer stages (120). For endometrial cancer, the new-onset diabetics (<5 years) have a two-fold risk compared with those with long-standing diabetes (≥5 years) (121). Besides, diabetic individuals with liver cancer and bladder cancer are associated with a poor survival compared with non-diabetic subjects (122).

#### Other Complications

Recent studies have regarded Alzheimer's disease as type 3 diabetes because of insulin signaling pathways damage (123). Moreover, sexual dysfunction becomes more and more popular in both men and women with diabetes (124). Besides, high prevalence of depression as one of psychological complications that result from diabetes, makes it harder for diabetics to manage blood glucose, thus leading to further complications (125).

## Diagnosis of Sarcopenic Obesity and Diabetes

### Diagnosis of Sarcopenic Obesity

There is currently no consistent diagnosis of SO, nevertheless an adequate one should include the individual diagnosis of obesity and sarcopenia. According to the criteria of WHO, BMI ≥ 30 kg/m^2^ or wrist circumference (men ≥ 102 cm and women ≥ 88 cm) is considered as obesity. However, whether these criteria are appropriate for each individual has been questioned. Alternatively, cutoffs of BF% (Body Fat Percentage) or other adiposity indices have been regarded as useful outcomes measure of obesity (126, 127).

European Working Group on Sarcopenia in Older People (EWGSOP) has proposed that (1) low muscle mass; (2) low muscle strength; (3) low physical performance are the three important parameters to define sarcopenia (128). Various techniques emerge for assessing muscle mass, among which, computed tomography (CT) and magnetic resonance imaging (MRI) are deemed as the gold standard to distinguish fat from other soft tissues, thereby, effectively estimating fat mass and muscle mass. However, it is hard to generalize CT and MRI due to the high cost, and the risk of radiation (for CT) (129). Therefore, another relatively inexpensive and low radiation method called dual-energy-X-ray absorptiometry (DXA) is recommended to estimate the lean and fat mass of the whole body or certain regions of body. Moreover, it also manifests strength in assessment of bone mass and density, thus simultaneously providing the conditions of bone, muscle, and fat (130, 131). In addition, as an affordable and available tool, bioelectrical impedance analysis (BIA) is used to measure muscle mass as well, whereas, the inaccuracy makes it unrecommended to diagnose sarcopenia (132). For it indirectly reflects body composition by evaluating the entire body and segmental reactance as well as resistance influenced by fluid retention and disease-related conditions. Hence, an overestimation is accompanied with a poor distinction between extracellular and intracellular fluid (133). Furthermore, air displacement plethysmography (ADP) measures body volume and body density and hence, total fat and lean tissue (134). In spite of the widespread use of anthropometric measurements, such as mid-upper arm circumference, calf circumference, and skin fold thickness, they are inaccurate (135). Muscle strength can be assessed by handgrip strength, knee flexion/extension, and peak expiratory flow. Handgrip strength is a great predictor of extremity muscle power and mobility. Furthermore, keen flexion/extension is strongly related with certain functional activities. Although peak expiratory flow manifests the strength of respiratory muscles, it is not considered as a separated method. Physical performance is defined by short physical performance battery (SPPB), usual gait speed, timed get-up-and-go test, and stair climb power test, which evaluate an individual's balance, gait, strength and endurance (136, 137).

In 1998, Baumarterner et al. (138) first defined sarcopenia as ASM divided by body height squared [ASM(kg)/height^2^ (m^2^)] two or more standard deviations below the mean of a young reference values measured by DXA. Later, Janssen et al. (139) proposed a definition of sarcopenia that the skeletal muscle mass index [skeletal muscle mass (kg)/weight (kg) × 100] is one or two standard deviations below the reference value of younger, healthy individuals measured by BIA. Then, Newman et al. (140) came up with a new criteria for sarcopenia according to appendiculate lean mass (ALM) adjusted for height and fat mass using residuals from linear regression models.

Based on the abovementioned assessments, the calculation of Baumarterner et al. with the addition of the BF% exceeds 60% of population of the peers is one definition of SO (141). Another definition includes that BF% exceeds 60% of population of the peers and muscle mass is inferior to 60% of population of the peers (142). However, the diagnosis of SO varies with the changes of the diagnosis of obesity and sarcopenia. After 2010, when the standard of sarcopenia was updated by EWGSOP, the diagnosis of SO should change too. The evaluation of muscle strength and physical function are supposed to be combined with the assessments mentioned above (4).

### Diagnosis of Diabetes

According to American Diabetes Association (ADA), there are four approaches to diagnosis diabetes: Fasting plasma glucose test (FPG), Two-Hour Oral Glucose Tolerance Test (OGTT), Glycated Hemoglobin (A1C), and Random plasma glucose coupled with symptoms (67). Table 1 summarizes these approaches as well as their respective diagnostic standards.

**Table 1:** Four approaches to diagnose diabetes according to ADA.

**Diagnostic tests**	**Diabetes diagnostic standards**
FPG	>126 mg/dL (7.0 mm/L)
OGTT	>200 mg/dL (11.1 mmol/L)
A1C	>6.5% (48 mmol/mol)
Random plasma glucose coupled with symptoms	>200 mg/dL (11.1 mmol/L)

#### Fasting Plasma Glucose Test (FPG)

The test should be carried out after at least 8 h fast, the level of FPG more than 126 mg/dL (7.0 mm/L) is considered as diabetes (67).

#### Two-Hour Oral Glucose Tolerance Test (OGTT)

In this test, individuals should consume at least 150 g per day of carbohydrate for 3–5 days without taking any medications. Then, the test is carried out before and 2 h after consuming 75 g of glucose. The 2-h plasma glucose is more than 200 mg/dL (11.1 mmol/L) is consistent with the diagnosis. Although OGTT is a standard test, it is less convenient and affordable than FPG (67).

#### Glycated Hemoglobin (A1C)

A1C shows the average of blood glucose over last 2–3 months, and it is relative accurate since less influence by acute illness or stress. Diabetes is diagnosed if the result of A1C is more than 6.5% (48 mmol/mol) (67).

#### Random Plasma Glucose Coupled With Symptoms

In patients with classic symptoms: polydipsia, polyphagia, and polyuria, random plasma glucose more than 200 mg/dL (11.1 mmol/L) is diagnosed as having diabetes (67).

## Treatments for Sarcopenic Obesity and Diabetes

### Conventional Treatments for Sarcopenic Obesity and Diabetes

The most common treatments of SO and diabetes are dietary intervention and physical activity. Although there are various approved and effective drugs for diabetes, there is currently no recommended pharmacological intervention for SO, not even for diabetics with SO.

#### Dietary Intervention for Sarcopenic Obesity

The main dietary interventions for SO consist of caloric restriction, protein intake, and micronutrient supplementation. It is noted that lack of proper randomized clinical trials (RCTs) makes expert opinions to be the major guidelines at present of dietary interventions for SO.

Caloric restriction aims to lose weight, inducing body composition changes, and a reasonable weight loss goal should be under 5–8% of the initial body weight (1, 143). As acute caloric restriction could promote proteolysis and negatively affect muscle protein synthesis, resulting in a further reduction of muscle mass, acute caloric restriction is not recommended. In contrast, chronic caloric restriction might increase muscle protein synthesis rather than downregulate (144, 145). Major RCTs in non-sarcopenic women with obesity have suggested that caloric restriction (500 kcal deficit) with at least 0.8 g/kg of protein intake can effectively lose fat and improve physical function (146, 147). Moreover, another study has demonstrated that even caloric restriction might cause loss of muscle mass, and hence, it is still more beneficial to mobility and strength when accompanying with resistance training (148).

Increased dietary protein can prevent weight loss-induced sarcopenia. According to existing evidence, a healthy young individual should take an average daily protein intake of 1 g/kg per day. While in older people, a higher protein intake should be provided in order to promote muscle protein synthesis due to anabolic resistance. Therefore, adequate protein intake of 1–1.2 g/kg per day is recommended for geriatrics, and an even higher intake for older people suffering from SO or other similar diseases. Acceptable protein intake of 1.2–1.5 g/kg per day should be provided for individuals with acute or chronic diseases, which are related to unbalanced body composition. Furthermore, individuals with critical illness or severe malnutrition need an increased protein intake from 2 g/kg per day (149–151). Moreover, oral protein supplements should be considered when ample dietary intake is not practical. A study assessed the effect of a diet moderately rich in proteins on lean mass in SO older women, and has indicated that sufficient protein intake is able to preserve muscle mass in older women with SO (18). A similar result has been shown in another study that high-protein diet improves muscle strength in SO patients and prevent weight loss-induced sarcopenia (152).

Despite that vitamin D supplement has not been properly tested in patients with SO, several studies have suggested that vitamin D deficiency is associated with lower muscle strength, greater body instability, falls and disability in geriatric population (153). Therefore, in order to minimize the adverse effects of weight loss, it may be necessary to increase vitamin D intake (154, 155). Besides, studies have elucidated that vitamin D has the capability of regulating bioactive metabolites, and thus, improving muscle function (156). A study has verified that 25-hydroxyvitamin D3 can indirectly impact muscle function as its free metabolite is more closely related to body fat than muscle gene expression (155).

#### Dietary Intervention for Diabetes

Dietary intervention plays an important role in the management of diabetes and diabetes-related complications. The intake of low glycemic food including cereal fiber or a mixture of whole grain and bran can decrease 18–40% of risk for diabetics (157). Moreover, the risk of diabetes reduces by 26% due to the consumption of one sugar-containing beverage more than 1 month compared to one per day (158). Furthermore, a systemic review of randomized clinical trials has assessed the effects of low carbohydrate, macrobiotic, vegan, vegetarian, Mediterranean, and intermittent fasting diets, compared to low-fat diets on diabetes control and management. However, there were no evident differences of low carbohydrate diets and low-fat diets in glycemic control, weight and lipids. The macrobiotic and vegan diets were beneficial to glycemic control, while the vegetarian diet demonstrated better weight loss and insulin sensitivity. Besides, the Mediterranean diet showed a greater regulation of A1C levels. Therefore, the study has concluded that vegan, vegetarian and Mediterranean diets are better strategies to control glycemic marker in diabetics (159). In addition, a study has also found that the benefits of Mediterranean diets were greater than low-fat diets in diabetic retinopathy, but no significant differences were found in diabetic nephropathy (160). Furthermore, dietary intervention is an important modifiable factor to reduce the incidence of cardiovascular diseases in diabetics (161). Hence, it may be necessary to implement dietary intervention in public health managements of diabetes.

#### Physical Activity for Sarcopenic Obesity

A combination of physical activity and dietary intervention is a more effective strategy to treat SO. Indeed, there are multiple biological effects of physical activity: promote insulin sensitivity, improve anabolic response to endogenous amino acids, activate skeletal muscle satellites cells and trigger the proliferation and differentiation of them, amplify irisin production, adjust hormonal milieu, increase mitochondrial biogenesis, ameliorate inflammation and reduce oxidative stress (162–164). Even though various studies have confirmed that exercise has positive effects for SO patients, many professional organizations recommend that a combination of resistance training and aerobic training seems to be the most practical way to improve physical performance (165, 166). For geriatric people, the main goal of physical training is to ameliorate elasticity, strength, and physical endurance. Meanwhile, as resistance training can improve flexibility, muscle strength, and muscle hypertrophy, aerobic training focuses on increasing physical endurance of older people (166). Therefore, two non-consecutive sessions of resistance training coupled with at least 150 min per week of aerobic training is recommended for all older individuals (167, 168). A meta-analysis involving 14 RCTs and a quasi-experimental trial has clarified that exercise, particularly resistance training plays a key part in improving body composition and physical performance in patients with SO (169). Also, an RCT, allocating 60 men and women to four groups (resistance training, aerobic training, combination training, and control), has found that the skeletal muscle mass, body fat mass, ASM/weight % and visceral fat area of treatment groups exhibited better results than control group. Noteworthy, the grip strength and knee extensor performance of resistance group were superior to those of the other groups (170). Furthermore, a clinical study evaluated the effects of resistance training, aerobic training, or combination training in obese older people. It has revealed that the physical performance test score was the highest in the combination group (21% increase) compared to the resistance group (14% increase) and aerobic group (14% increase). But strength increased more in the resistance group (19% increase) than in the combination group (18% increase) and aerobic group (4% increase). While peak oxygen consumption (milliliters per kilogram of body weight per minute) increased most in the aerobic group (18% increase) compared to the combination group (17% increase) and resistance group (8% increase). Thus, resistance training combined aerobic training is the most effective way to alleviated physical disability (171).

#### Physical Activity for Diabetes

Several studies have demonstrated that physical activity is an effective regimen to lower the risk of developing diabetes by improving the β-cell function (172). A randomized controlled trial has evaluated the effects of moderate to intense exercise on pancreatic fat content and β cell function. The amount of pancreatic fat decreased in both healthy group (from 4.4 to 3.6%) and prediabetes/T2D group (from 8.7 to 6.7%), without significant differences observed for the improvement of β-cell function in different exercise strategies (173). Another study also has confirmed that high-intensity interval training is strongly associated with the improvement of β-cell function in patients with T2D. The total body-fat percentage was reduced, whereas lean body mass was protected. Noteworthy, high-intensity interval training was not able to regulate the levels of fasting plasma glucose, insulin, C-peptide, pro- insulin, and free fatty acids, nor did the levels of first-phase (0–30 min) and late-phase (30–180 min) plasma glucose, insulin, C-peptide, and proinsulin (174). Furthermore, as mentioned previously that the combination of resistance and aerobic training is beneficial to SO, it is a highly effective strategy for diabetes as well. A randomized controlled trial conducted in America has examined the effects of resistance training alone, aerobic training alone, and a combination of both on A1C within diabetics. Compared to control group, there were no significant mean changes of A1C in either the resistance training alone group (−0.16%; 95% CI: −0.46–0.15%; *P* = 0.32) or the aerobic training alone group (-0.24%; 95% CI: −0.55–0.07%; *P* = 0.14), however, there was a significant absolute mean change of A1C in the combination training group. Moreover, the combination training group had a maximal progression of oxygen consumption compared to other groups. All three exercise groups decreased waist circumference by 1.9–2.8 cm as compared to the control group. Therefore, in spite of the benefits of resistance training alone and aerobic training alone on diabetics, the combination of both could improve the level of A1C, which could hardly be obtained by each training exercise alone (175).

#### Pharmacological Intervention for Sarcopenic Obesity and Diabetes

In spite of many novel pharmacological interventions are under investigation, such as testosterone supplement, selective androgen receptor modulators, and myostatin inhibitors, there is currently no approved drugs for SO (176–178). However, there are approved and effectively drugs for diabetes. Metformin is considered as one of the most prevalent medications for diabetes management (179). Based on existing evidence, the National Institute for Health and Care Excellence (NICE) guidance for adults with type 2 diabetes recommends standard-release metformin as the initial drug treatment (180). A considerable number of studies have also found that metformin decreases fasting plasma glucose (PG) concentration and hemoglobin A1c by conserving the β-cell function or by decreasing liver glucose production (hepatic gluconeogenesis) (181–184). Also, thiazolidinediones can promote insulin sensitivity, increase glucose metabolism, and preserve the β-cell function through activating PPAR-γ (185, 186). Meanwhile, they can reduce plasma free fatty acid and intramyocellular lipid content to increase insulin sensitivity and redistribute fats from visceral to subcutaneous adipose to alleviate diabetes. Pioglitazone and troglitazone, belonging to thiazolidinediones, have the effect of controlling the progression on gestational diabetes (187, 188). Besides, α-Glucosidase Inhibitors contribute for prolonging the overall carbohydrate digestion duration and reducing the rate of glucose absorption (189). Whereas, it should be noted that they do not increase insulin sensitization (190), and a systematic review disapproves of acarbose dosages higher than 50 mg (three times a day), as there is no better effects on glycated hemoglobin (191). Therefore, α-Glucosidase Inhibitors should not be applied as initial drugs, and it is more positive to combine them with other types of anti-diabetic drugs. Moreover, incretins can shrink appetite, thus reduce food intake, leading to weight reduction (192). Because of the impact on weight reduction, incretins may find increasing use in diabesity, which is the coexistence of diabetes and obesity (193). In addition, Sodium-Glucose Cotransporter (SGLT) 2 Inhibitors are one of the latest pharmacological interventions to decrease the reabsorption of glucose in kidney and lower FGP and A1C, which enhances urinary glucose elimination and attenuates blood glucose level. Meanwhile, they can also positively affect cardiovascular diseases due to sodium decrease, uric acid absorption, and blood pressure reduction (194).

### Complementary and Alternative Treatments for Sarcopenic Obesity and Diabetes

#### Herbal Medicine and Derivative

With the growing of popularity of herbal medicine, many studies have indicated that herbal medicine or related derivatives may be effective methods to treat SO and diabetes. A study has reported two cases about using wild ginseng complex (WGC) on two patients who only wanted to lose abdominal fat, but not in other parts of body. After 3 weeks of WGC intervention, the two patients had an increase in muscle mass, protein content, and basal metabolic rate. Therefore, WGC intervention may be a new alternative treatment for age-related sarcopenic obesity but more studies using larger samples are required to support this (195). In another study, three major herbal medicine including Zuo Gui Wan, red raspberry leaves, and Orthosiphon stamineus were found beneficial to gestational diabetes by controlling glucose (196). Hence, effective management of diabetics with SO using herbal medicine and derivatives may be plausible but more supportive evidences are still required.

#### Acupuncture

Acupuncture has been used in diabetes for a long time in Asian countries and recent studies have also suggested that acupuncture may alleviate SO (197–199). A randomized controlled trial, using electrical acupuncture coupled with essential amino acid supplementation to treat SO in male older people, has indicated that both electrical acupuncture with oral essential amino acids group and oral essential amino acids alone group can decrease BF% and increase ASM/H2, with the combination group being more effective than another group. Moreover, the combination group can increase muscle mass in a shorter time (197). Besides, a meta-analysis of randomized controlled trials has confirmed that acupuncture should be recommended as a complementary treatment in T2D control, particularly with obesity or other metabolic disorders (195). Although the underlying mechanism of acupuncture on diabetes remains unclear, studies have suggested that it may be related to adjust nerve conduction, modulate signal pathways, regulate hormonal level, and ameliorate oxidative stress level (200). Further investigations are required to prove that acupuncture is indeed effective for the treatment of patients with diabetes and SO.

## Discussion

The occurrence of SO and diabetes has a rapid growth worldwide because of lifestyle changes and longer life expectancy. Indeed, they share many common risk factors, especially in aging and obesity. Moreover, a study has found that in the case of similar BMI, diabetics have decreased lean body mass and increased body fat mass compared with non-diabetics (57), indicating that diabetes is associated with increased risk of SO. Although the underlying mechanism of the association remains unclear, we speculate a bidirectional interplay in obesity, low-grade inflammation, insulin resistance and sarcopenia. SO combines sarcopenia and obesity, and low-grade inflammation plays a crucial role in the pathogenesis of diabetes (11). Therefore, SO may have synergistic effect with low-grade inflammation to exacerbate insulin resistance, further impairing glucose metabolism. Noteworthily, physical activity is helpful for both SO and diabetes. However, it should be treated with caution due to the degeneration of muscle mass for SO and venerable feet for diabetes. The young individuals in good metabolic control are able to do most activities, but the middle-aged and older individuals or patients with other complications are encouraged to check with the doctor to avoid injuries of intensive exercise (201). Generally, for patients with SO and diabetes, an appropriate warm-up and cool-down period should be included before and after physical activity session. A short warm-up session at a low-intensity level helps skeletal muscles, heart, lungs prepare before formal exercise. After the physical activity session, a short cool-down session should be conducted similarly as the warm-up session to gradually bring the heart rate down. Proper stretches should be structured after warm-up and cool-down period to protect muscles. Moreover, suitable footwear should be emphasized to prevent blisters and keep the feet dry because it is easy to cause trauma of the feet for diabetics. Fluid intake affects blood glucose levels and heart function, thus, during physical activity, fluid should be taken early and regularly. Finally, the diabetics should never forget to test blood glucose regularly (202, 203).

Although pharmacological intervention has been widely used in treating diabetes, doctors and patients need to be cautious of the side effects of the drugs. Metformin has been reported to cause deficiency in vitamin 12 and folic acid. Thiazolidinediones can cause bladder cancer and fractures, and combined insulin-thiazolidinediones therapy may lead to heart failure. The common adverse reactions of SGLT2 inhibitors are urinary tract infections, increase in low-density lipoprotein (LDL) cholesterol, bone fractures, and they may sometimes cause ketoacidosis. Therefore, pharmacological intervention needs to be monitored, especially in elderly patients and patients with other complications (204). Additionally, there are some limitations in the complementary and alternative treatments. Although several studies have revealed some positive results, the overall quality of the evidence is low, and the available data are too few to adequately suggest that herbal medicine and derivative and acupuncture are useful. Thus, more large-scale, multicenter, high-quality RCTs are required in the future, which will lead to deeper understandings of complementary and alternative treatments for SO and diabetes. Furthermore, it is important to raise the awareness of the high prevalence of the sarcopenia in the obese population, which seems to be strongly associated with diabetes, and screening for SO in subjects with obesity during clinical practice may be necessary. Therapies for SO and diabetes should be treated carefully and personally in order to minimize adverse effects.

## Conclusion

By analyzing research evidence from previous studies, this review identified possible pathogenesis of SO and diabetes, such as malnutrition, insulin resistance, low-grade inflammation, and hormonal changes. Meanwhile, the underlying mechanisms of insulin deficiency and insulin resistance have been discussed to provide a better understanding of the association between SO and diabetes. Also, complications of SO and diabetes have been explored to urge more attention on SO and diabetes. Additionally, we have consolidated the novel diagnostic methods of SO and diabetes. Furthermore, different treatment options have been exhibited that dietary intervention and physical activity are considered as prevalently effective treatments for diabetics with SO, and some complementary and alternative interventions have shown some positive effects on SO and diabetes. As a final suggestion for the prevention and treatment of diabetes and SO, we recommend dietary intervention and regular exercises, coupled with specific drugs prescribed to individuals by the clinicians.

## Author Contributions

ZL: conceptualization. YT and PW: resources. MW: writing—original draft preparation. YS: figure preparation. ZL, YT, XW, and PW: writing—review and editing. ZL and PW: supervision. PW: project administration. YT and PW: funding acquisition. All authors have read and agreed to the published version of the manuscript.

## Conflict of Interest

The authors declare that the research was conducted in the absence of any commercial or financial relationships that could be construed as a potential conflict of interest.
